# Reply to M. Swanson et al

**DOI:** 10.1200/JGO.17.00049

**Published:** 2017-07-17

**Authors:** Jose Jeronimo, Sarah Temin, Philip E. Castle, Surendra S. Shastri

**Affiliations:** **Jose Jeronimo**, Seattle, WA; **Sarah Temin**, American Society of Clinical Oncology, Alexandria; **Philip E. Castle**, Global Coalition Against Cervical Cancer, Albert Einstein College of Medicine, Arlington, VA; and **Surendra S. Shastri**, Tata Memorial Center, Mumbai, India

We appreciate the insight and comments of Swanson, Gimei, and Huchko^1^ in their correspondence regarding *Journal of Global Oncology* publication “Secondary Prevention of Cervical Cancer: ASCO Resource-Stratified Clinical Practice Guideline.”^2^ We welcome their appreciation of this ASCO resource-stratified guideline, which supports the idea that every woman around the globe should have access to affordable and effective cervical cancer screening. The ASCO Expert Panel Steering Committee feels this letter helps amplify the guideline.

Swanson et al^1^ state that “ASCO recommends VIA [visual inspection with acetic acid] scale-up in settings where HPV [human papillomavirus] testing is considered not feasible, as a necessary step to create infrastructure for future HPV testing. We disagree with this recommendation,” in reference to the ASCO recommendation (only in the basic setting) that “if HPV DNA testing for cervical cancer screening is not available, then VIA should be offered with the goal of developing health systems and moving to population-based screening with HPV testing at the earliest opportunity.”^2^ Swanson et al state that “given the increasing availability of feasible, acceptable HPV DNA tests which can be self-collected by women outside a clinic, we suggest that resources may be better spent developing community-based HPV testing for primary screening, rather than scaling up widespread VIA.” The ASCO recommendation was based on a review of evidence on HPV testing and VIA. It recognizes that in some basic settings, there is little infrastructure (eg “1% or less in Bangladesh, Ethiopia, and Myanmar”^3^) for public health systems with sufficient development to have outreach workers, and it strives to make recommendations both for current situations and for what planners could put in place in future. The guideline states that research on VIA has shown mixed results and that “the goal [is] moving to population-based screening with HPV testing at the earliest opportunity.”^2^ In addition, the guideline reviews emerging literature on self-collection and largely agrees with the potential use of it in some settings; ASCO will update the guideline pending additional evidence on self-collection.

In addition, the letter by Swanson et al^1^ suggests that implementing the ASCO recommendation that “women who are postpartum should be screened with VIA 6 weeks after delivery in basic settings”^2^ is “missing a key opportunity to interact with an at-risk population.” The ASCO recommendation was the result of extensive expert panel discussion, and because of similar concerns (“in some settings, loss to follow-up may be a concern”^2^), this was a formal consensus-based (rather than evidence-based) recommendation (designated as “Evidence quality: insufficient; Strength of recommendation: weak.”^2^). The guideline authors believe this provides flexibility in settings where women are less likely to have postpartum visits, as Swanson et al describe for Uganda; the target age for the recommendation for basic (as well as limited and enhanced) settings is women age 30 years or older, and pregnancy is not as common after age 30 years in basic settings as it is for women living in maximal resource settings, who are more likely to access services postpartum. We do agree that “we need to create strategies linking the post-partum cervical cancer screening to other health visits” (Jose Jeronimo, personal communication) and with the statement by Swanson et al “optimizing use of existing infrastructure will be essential to effective national screening programs, especially in basic settings with competing health priorities.”
